# Presynaptic actin nanostructures: A reproducibility case study

**DOI:** 10.1111/jmi.70138

**Published:** 2026-07-24

**Authors:** Rensu P. Theart, Florian Levet, Paul Hernández‐Herrera, Christophe Leterrier, Laura R. de la Ballina

**Affiliations:** ^1^ Department of Electrical and Electronic Engineering Stellenbosch University Stellenbosch South Africa; ^2^ Interdisciplinary Institute for Neuroscience, UMR 5297 University of Bordeaux, CNRS Bordeaux France; ^3^ Bordeaux Imaging Center, University of Bordeaux, CNRS, INSERM Bordeaux France; ^4^ Facultad de Ciencias Universidad Autónoma de San Luis Potosí San Luis Potosí México; ^5^ Institut de Neurophysiopathologie, UMR7051, NeuroCyto Aix Marseille Université, CNRS Marseille France; ^6^ Faculty of Medicine Centre for Cancer Cell Reprogramming Institute of Clinical Medicine University of Oslo Montebello Oslo Norway; ^7^ Department of Molecular Cell Biology Institute for Cancer Research The Norwegian Radium Hospital, Oslo University Hospital Montebello Oslo Norway

**Keywords:** actin nanostructures, bioimage analysis, GloBIAS, presynapses, reproducibility

## Abstract

In an effort to assess the reproducibility of bioimage analyses in current publications, we took part in a Global BioImage Analysts’ Society (GloBIAS) initiative to try and reproduce results from published articles. We attempted to reproduce core findings from Bingham *et al.* (2023), who investigate the actin organisation in presynaptic structures by using diffraction‐limited and super‐resolution microscopy. While the original paper unveiled novel biological insight, it lacked sufficient detail in the bioimage analysis methodology, limiting the depth of reproducibility we could achieve. Through frequent contacts with the corresponding author, we were able to replicate qualitative aspects of the analysis of actin nanostructures in bead‐induced presynapses. We performed image reconstruction from super‐resolution microscopy data, automatic image registration and visual inspection, followed by manual annotation of structures of interest in approximately 35 images. From this exercise, we provide concrete evidence that key practises such as sharing of example datasets, depositing manual annotations and documenting manual decision criteria and consensus procedures are essential for making bioimage analysis workflows reproducible. Our experience further highlights that transparent data sharing, adherence to bioimage analysis standards, and close collaboration between experimentalist and bioimage analysis specialists are critical to ensure the reproducibility of today's complex biological imaging studies.

## 1 INTRODUCTION

As part of the Global BioImage Analysts’ Society (GloBIAS) first in‐person workshop (*GloBIAS Bioimage Analysts Workshop*, 5–8 November 2024, Gothenburg, Sweden), we conducted a practical exercise addressing ‘Bioimage analysis workflows’, where workshop participants were divided into working groups and assigned published articles where bioimage analysis workflows had been used.^1^ The assignment consisted of trying to reproduce, based on the *Methods* described in the paper, the published bioimage analysis workflows and presenting the results during a dedicated session in the GloBIAS workshop with the final aim of assessing how reproducible bioimage analysis workflows are at present (Figure 1).

**FIGURE 1:**
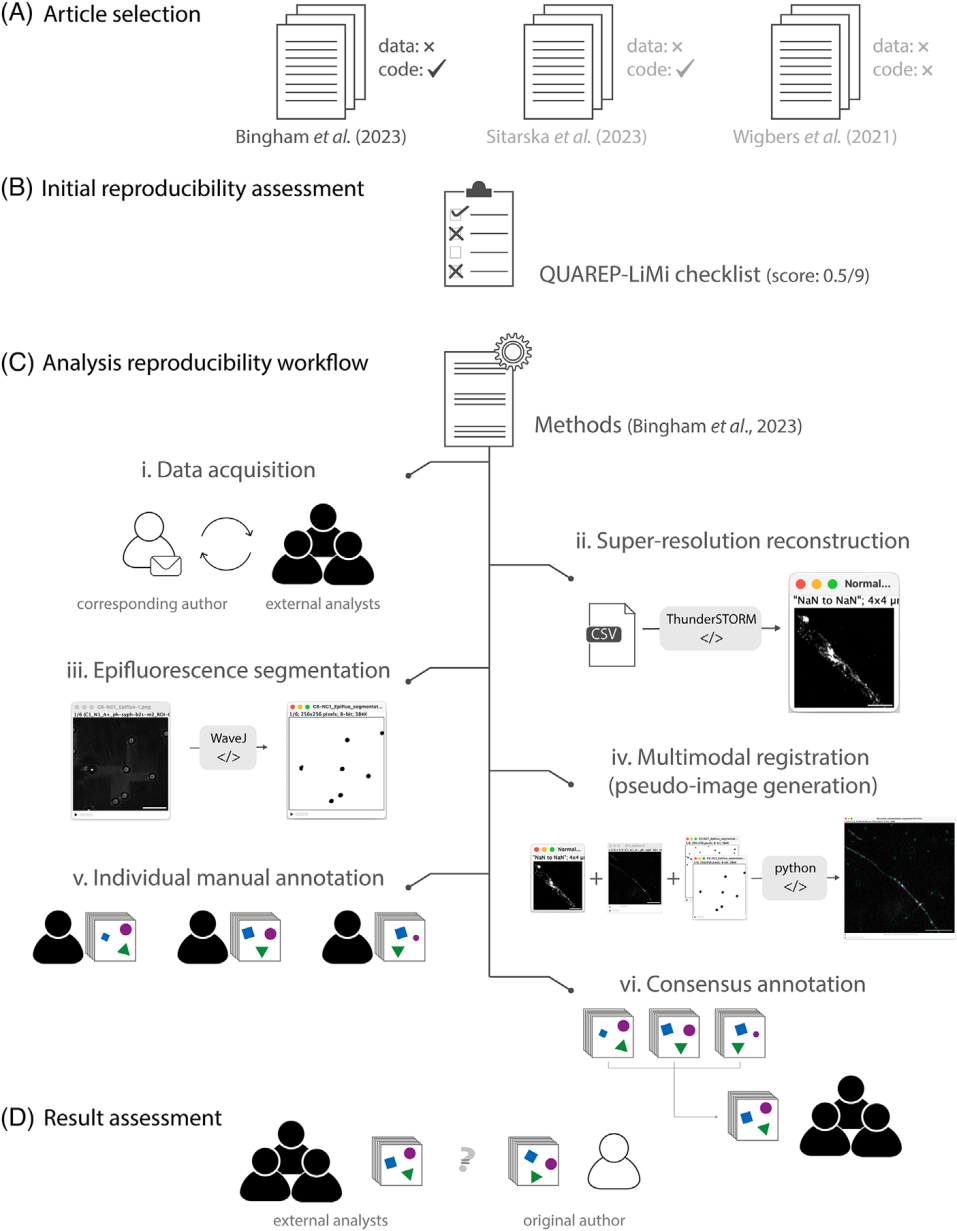
Reproducibility workflow. Overview of the reproducibility workflow performed as part of the *GloBIAS Bioimage Analysts Workshop* (5–8 November 2024, Gothenburg, Sweden). (**A**) Prior to the workshop we were assigned research articles that reported bioimage analysis workflows. The exercise consisted in assessing the reproducibility of these workflows using the information provided in the Methods section of the original publications. (**B**) We used the checklist developed by members of QUAREP‐LiMi as an initial assessment of reproducibility. (**C**) We then attempted to reproduce the analysis workflow of Bingham *et al.* (2023). To do so, we (**i)** contacted the corresponding author of the original publication to obtain sample image data (∼35 ROIs), (**ii)** performed SMLM data reconstruction, (**iii)** segmented epifluorescence images, (**iv)** automatically registered SMLM, epifluorescence and segmented images, and (**v)** manually annotated the images (each external analyst individually). Finally, we (**vi)** derived a consensus annotation. (**D)** That consensus was compared to the original annotations provided by the authors of Bingham *et al.* (2023) for those same ROIs.

In recent years the bioimage analyst community has generated guidelines to follow when publishing bioimages and bioimage analysis workflows, in hope of increasing the rigor and reproducibility of published images and corresponding extracted data and with the aim of producing scientific data more aligned with the FAIR principles.^2^, ^3^, ^4^ For this exercise, we used the ‘*Checklists for publication of image‐analysis workflows*’ included in Schmied *et al.* (2024) as a reference framework to assess the reproducibility of assigned workflows.^2^ It is important to note that the articles we assessed were published before the community checklists were released. By applying these checklists retrospectively, we aimed to illustrate how following such emerging standards could have supported our reproduction attempts, and how their broader adoption could strengthen the reproducibility of future bioimage analysis workflows.

After two unsuccessful attempts (due to lack of access to original data from other articles), we assessed the reproducibility of the work published by Bingham *et al.* (2023) which investigates the organisation of actin nanostructures in presynapses.^5^ In this paper, the authors exploit a previously validated model where presynapses are induced in cultured neurons using polylysine‑coated beads.^6^ By studying the induced presynaptic structures using Single Molecule Localisation Microscopy (SMLM), the authors identify three distinct actin nanostructures in presynapses: actin mesh, actin rails and actin corrals, which they further confirm to be present in natural presynapses.

Light microscopy has profoundly advanced our understanding of cell biology. Thanks to efficient fluorescent probes and robust labelling protocols, fluorescent microscopy enables the direct visualisation of proteins‐of‐interest with high specificity and excellent contrast, greatly expanding our ability to monitor cellular mechanisms in fixed and living tissues. Nevertheless, due to the wave nature of light, the resolution of conventional light microscopy is limited to approximately half the wavelength of the emitted light, that is, ∼200 nm at best. As a result, standard wide‐field or confocal microscopy cannot resolve the fine structural organisation of biological samples below this limit. Within cells, and especially in neurons, many structures are organised at spatial scales well below this resolution barrier.

Over the last decade, several approaches have been developed to circumvent the diffraction limit, prompting a major revolution in light microscopy. Among these, single‐molecule localisation microscopy has transformed cell biology by enabling the observation of fluorescent molecules in living cells with spatial resolution of just a few nanometres.^7^ By optically switching different subpopulations of molecules between ‘on’ and ‘off’ states over time, this technique enables spatially separated molecules to be precisely localised by image analysis, typically achieving resolutions of 10–50 nm. This approach ultimately allows researchers to count and track biomolecules in their native cellular environment by directly analysing the positions of localised molecules over time.

Whereas the possibility of resolving structures below the diffraction limit is remarkable, super‐resolution images are reconstructed through complex, algorithm‐dependent processing steps (e.g. filtering, drift correction, rendering), where artefacts can be introduced.^8^ The use of different software, parameters, or thresholds can yield different structures from the same raw data. Therefore, having well‐documented, reproducible workflows is essential to distinguish genuine biological organisation from analysis artefacts and to ensure that the resulting biological conclusions are robust.

From the extensive work of Bingham *et al.* (2023) (the original article contains 10 main figures and 7 supplementary ones), we selected Figure 7 as the focus of our reproducibility exercise. In that figure, the authors combine SMLM and fluorescence microscopy to confirm the presence of *actin mesh, rails*, and *corrals* in various types of induced presynapses. Our task was to assess whether – based on the analysis steps and textual description published by the authors – we were able to identify the same structures they did.

After getting in contact with the corresponding author of the original publication and having access to sample image data and a more detailed description of the actin nanostructures of interest, we proceeded with our annotation exercise.

We were partially successful in following the author's instructions and in identifying actin nanostructures in bead‐induced presynapses, with discrepancies in the results likely due to the different backgrounds of each team member.

During this reproducibility exercise, we joined other community efforts to assess the reproducibility of bioimage analysis workflows. Whereas previous works have largely focused on benchmarking different solutions to a given bioimage analysis challenge, we instead attempted to reproduce a published analysis workflow, uncovering several limitations.^9^, ^10^, ^11^ By documenting both successful and unsuccessful steps in our reproduction attempt, we aim to highlight practical, community‐aligned recommendations that are easy to adopt when reporting analysis workflows. Our experience suggests that adhering to the emerging standards and guidelines of the bioimage analysis community could help mitigate many of the challenges we encountered and improve the reproducibility of future studies.

## 2 METHODS

### 2.1 Contacting the corresponding authors to gain access to the original data

For all articles considered in this reproducibility assignment, the lack of directly available image and/or code data required contacting the corresponding authors of the original works (as stated in the ‘*Data and Code Availability*’ sections of the original publications) to obtain a dataset we could work with (Figure 1A).

In the case of the work by Bingham *et al.*, after contacting the corresponding author we gained access to sample SMLM image data – 34 regions of interest (ROIs) from 3 replicates (NC1, NC2 and NC3) – and the authors’ annotations for those same ROIs (which we only consulted to compare results once we had blindly performed our analysis) first and the corresponding epifluorescence images later on (as we realised we were missing some relevant information we could not extract from only the SMLM images). Note that the author‐provided ROI annotations we obtained corresponded to those made by a single author, rather than the final annotations presented in Figure 7 of the original paper. We have now made all images publicly available through Zenodo (https://doi.org/10.5281/zenodo.20386408).^12^, ^13^

Several rounds of communication with the corresponding author were required to get clarifications regarding the analysis steps, as well as the definitions of the different presynaptic actin nanostructures we needed to identify (Figure 1C, i).

Due to the essential role of the corresponding author of the original publication in enabling the reproducibility of the bioimage analysis workflow evaluated, they are included in this work's author list. We wish to state that this inclusion did not affect our assessment of the reproducibility of the original work.

### 2.2 SMLM acquisition and image reconstruction

Details of SMLM acquisition are provided in the *Methods* section of the original work by Bingham *et al.* ^5^ Here, we highlight the parameters most relevant for reproducible reconstruction of the super‐resolution images from the localisation files shared by the authors (available at https://doi.org/10.5281/zenodo.20386408, under *00_raw_images*, within the *Locs_TS_ROIs* subfolders).^13^

The data in Bingham *et al.* were generated over a period of approximately 5 years using two approaches: (i) the Nikon N‐STORM software, with direct export of coordinate files; and (ii) the deep‐learning‐based DECODE software, which was trained and applied as described in Speiser *et al.* ^14^ In all cases, STORM imaging of Alexa Fluor 647‐labelled samples was performed with a 647 nm laser operated at 100% of its 125 mW output (measured at the fibre output, no systematic measurements were available at the objective back aperture). For the Nikon pipeline, lateral drift during acquisition was corrected by cross‐correlation in the N‐STORM software. For the DECODE pipeline, 3D drift correction was performed by redundant cross‐correlation implemented in SMAP, following the algorithm of Wang *et al.* ^15^

As indicated in the ChriSTORM (version 1.3) GitHub repository (https://github.com/cleterrier/ChriSTORM) cited in the original work of Bingham *et al.*, image reconstruction from localisation coordinates was performed using the ThunderSTORM plugin for Fiji/ImageJ, in the fork maintained by Koen J.A. Martens (https://github.com/kjamartens/thunderstorm). ChriSTORM provides macros for converting, filtering, and batch‐processing localisation data and for generating reconstructed images, thereby complementing the ThunderSTORM plugin.^16^, ^17^

To ensure that images can be reconstructed reproducibly from the localisation files, we relied exclusively on the ThunderSTORM reconstruction module, independent of how the localisations were originally obtained (N‐STORM or DECODE). Because ThunderSTORM uses pixel coordinates internally for some functions, the only hardware parameter that affects reconstruction from pre‐fitted localisation coordinates is the effective camera pixel size. For these data, this value is 160 nm per pixel, corresponding to 16 µm physical pixels on the Andor iXon DU‐897 EMCCD camera imaged through a 100× objective. Other camera settings used during acquisition (15 ms exposure time, conversion gain 0.248 e^−^/ADU, and baseline 100 ADU) influence localisation precision at the fitting stage but do not enter the reconstruction when starting from finished coordinate files.

The coordinate files shared with us were in the ThunderSTORM format (column definitions described at https://www.neurocytolab.org/tscolumns/). In this format, each line corresponds to a single fitted localisation. The columns relevant for reconstruction are (*X*, *Y*, σ *X*, σ *Y*) for 2D‐STORM and (*X*, *Y*, *Z*, σ *X*, σ *Y*, σ *Z*) for 3D‐STORM. Theoretical localisation precision is encoded directly in the coordinate files via the σ (standard deviation) values reported for each localisation. For 2D‐STORM datasets processed with the Nikon N‐STORM software, a single lateral σ value is provided, estimated according to the Mortensen formula.^18^ For 3D‐STORM datasets processed with DECODE, the model jointly infers *X*, *Y*, and *Z* coordinates together with their respective standard deviations, which are written to the coordinate files. From these fields, average lateral and axial localisation precision can be extracted for each dataset. Experimental localisation precision was also characterised independently by localising sub‐diffraction fluorescent beads under imaging conditions matched to Alexa Fluor 647 single molecules. As reported in Leterrier *et al.* (2015),^16^ Figure S1, this yielded standard deviations of approximately 6 nm in *XY* and 13 nm in *Z* (the values in the figure are expressed as full width half maximum (FWHM), which is defined as 2.53·σ in that work). These values are sufficient to resolve the presynaptic actin nanostructures of interest, although the ultimate achievable image resolution also depends on localisation density (Nyquist sampling).

For reconstruction, we used Fiji (ImageJ2, version 2.14.0/1.54f) with ThunderSTORM.^17^, ^19^, ^20^ As shown in Figures 1C, ii and 2, localisation .csv files from the ‘*Locs_TS_ROIs*’ folders were imported with the camera pixel size set to 160 nm, and super‐resolution images were rendered using the ThunderSTORM *Gaussian reconstruction* in 2D mode (3D option disabled) with ‘*Auto size by results*’ for the ROI. All ROIs were reconstructed using identical settings, irrespective of the localisation‐fitting pipeline. The resulting super‐resolution renderings of actin localisation patterns are available at https://doi.org/10.5281/zenodo.20386408, under *01_processed_images* in the *Recs_TS_ROIs* folders for each replicate.^13^

**FIGURE 2:**
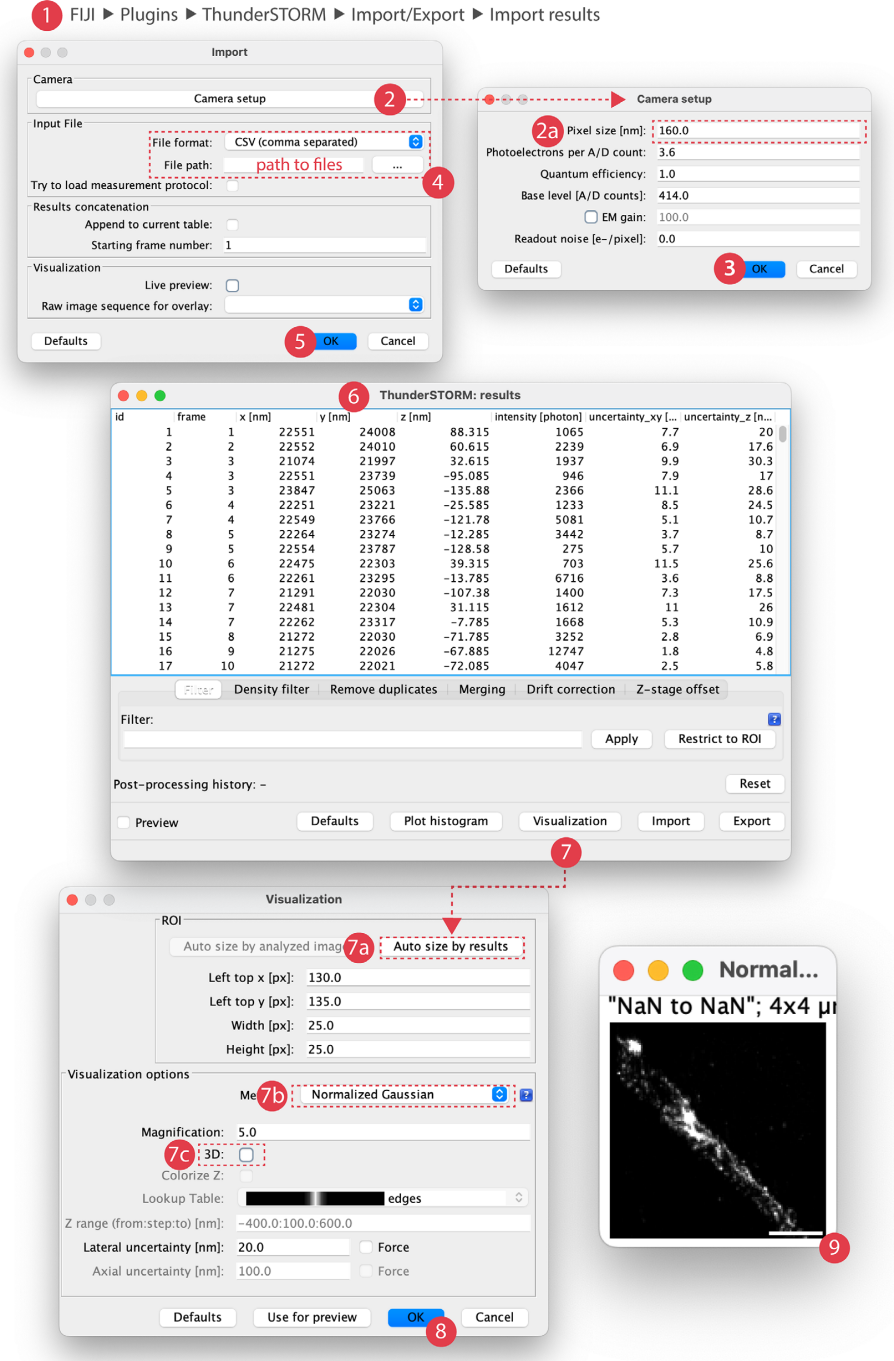
SMLM image reconstruction using the ThunderSTORM Fiji plugin. Sequential steps to use the ThunderSTORM Fiji plugin to reconstruct single molecule localisation microscopy images. After starting the plugin (1), we access the ‘Camera setup’ menu (2) to adjust the pixel size (in nm) (2a) and press OK (3). We then proceed to adjust the ‘Input file’ section, selecting *CSV (comma separated)* as File format and indicating the local path to the corresponding .csv files (4). Once this is in place we click OK (5) and are presented with the ThunderSTORM:results menu (6). We click ‘Visualization’ (7) and in the appearing window, we click ‘Auto size by results’ (7a) choose *Normalised Gaussian* method (7b) and untick the 3D box (7c). After clicking OK (8), we get the SMLM reconstructed image (9). In the figure, we present screenshots from Fiji. Scale bar  =  1 µm (the scale bar was added explicitly and is not part of the ThunderSTORM output).

### 2.3 Description of our structures of interest: presynaptic actin nanostructures

Our aim was to annotate the ROIs we had been provided for the corresponding structures of interest. It is worth noting that in the original publication these structures had been defined following an unbiased consensus process, where two specialists independently annotated ∼50 synapses, and then the authors defined as *typical* structures the ones that were frequently observed by both specialists. Thus, we followed the criteria defined by the authors (in the *Methods* section of the original paper, under the ‘STORM image analysis’) to identify the three types of presynaptic actin nanostructures: (a) *actin mesh*: a small cluster of actin opposed to the bead contact and within the presynaptic marker cluster, (b) *actin rails*: linear structures within the presynaptic marker cluster, and (c) *actin corrals*: large actin clusters at the periphery or just outside of the presynaptic marker cluster (depicted in Figure 4A).^5^ These criteria have subsequently been adopted in another study.^21^

Due to our lack of experience in the field, we had to confirm with the authors that the ‘presynaptic marker cluster’ corresponded to the synaptophysin label. We realised that we were missing those data, as well as information related to ‘bead contact’. We got back in contact with the authors, asking for extra sample image data we could extract that information from and were provided with fluorescent images for each replicate (which can be found at https://doi.org/10.5281/zenodo.20386408, under *00_raw_images* and each corresponding replicate folder: *NC1_*, *NC2_* and *NC3_epifluo.tiff* files).^13^

All external analysts involved in this study had prior experience with biological image analysis but were not domain experts in synapse biology. As such, identification of the structures of interest relied exclusively on the textual descriptions and limited example images provided in the original publication. These definitions were interpreted as operational guidelines; however, their qualitative nature introduced ambiguity in distinguishing between classes, particularly for analysts without specific biological expertise.

### 2.4 Automatic registration of epifluorescence and SMLM images and generation of multimodal pseudo‐images

After obtaining the epifluorescence images corresponding to the ROIs where they had performed SMLM acquisitions (actin STORM images), we could get information related to presynaptic marker cluster synaptophysin (channel 3 in epifluorescence images), axon marker ß2‐spectrin (channel 4 in epifluorescence images) and the presynaptic‐inducing beads (channel 6 in epifluorescence images).

Although a method describing epifluorescence image processing is provided in the original study, it was developed for a different type of quantification and was not directly applicable to our analysis. In our case, the epifluorescence channels were primarily used to identify structures surrounding or being part of the synapses and relate them to the presynaptic nanostructures observed in SMLM data. Because we are not specialists in neuroscience, interpreting epifluorescence and super‐resolution images separately would have been challenging. We therefore chose to register both modalities to a common spatial resolution and to segment the bead and synaptophysin signals in the epifluorescence channels, facilitating the interpretation of the multimodal data. This approach, while potentially differing from the original authors’ processing, allowed a more controlled and interpretable integration of modalities. For this purpose, we relied on wavelet‐based segmentation, a method well suited for detecting blob‐like structures across multiple spatial scales, and for which we had a robust, validated implementation readily adaptable to our workflow.

To segment both synaptophysin and bead markers, we used wavelet analysis with WaveJ (https://github.com/flevet/WaveJ), an update of the wavelet component of SpineJ (https://github.com/flevet/SpineJ) that was extended to handle image stacks.^22^, ^23^ WaveJ was installed in Fiji by adding the *wavej_package.jar* file from WaveJ release 1.0.0 (https://github.com/flevet/WaveJ/releases/tag/1.0.0) to the Fiji ‘*plugins*’ folder. Once the image (individual channel) containing the objects to segment was open, we run WaveJ. The plugin uses ‘à‐trous’ wavelet algorithm, which computes a series of multi‐scale wavelet coefficients by iterative convolutions of increasing kernels.^24^ It is then sufficient to filter those coefficients at the level corresponding to the size of the objects of interest to reliably segment them. For each marker, we empirically explored several combinations of wavelet levels, intensity thresholds, and minimum object sizes using WaveJ's live visual segmentation output and selected the parameters that yielded clean segmentations. For synaptophysin, we used the wavelet coefficient level 1 with a threshold of 5 and a minimum size for the segmented clusters of 10 pixels. For bead segmentation, we used the wavelet coefficient level 2. This time we had to slightly adjust the thresholds used depending on the signal to noise ratio and image quality of the different replicates. For thresholding the coefficients, we used two values: 10 for NC1, NC2 and NC3 images 1 to 8, and 3 for NC3 images 9 to 15. For the minimum size of the beads, we also used two values: 80 for NC1 and 60 for NC2 and NC3 (all images). These steps are illustrated in Figures 1C, iii and 3 (which exemplifies the bead segmentation on channel 6 of *NC1_epifluo.tiff*).

**FIGURE 3:**
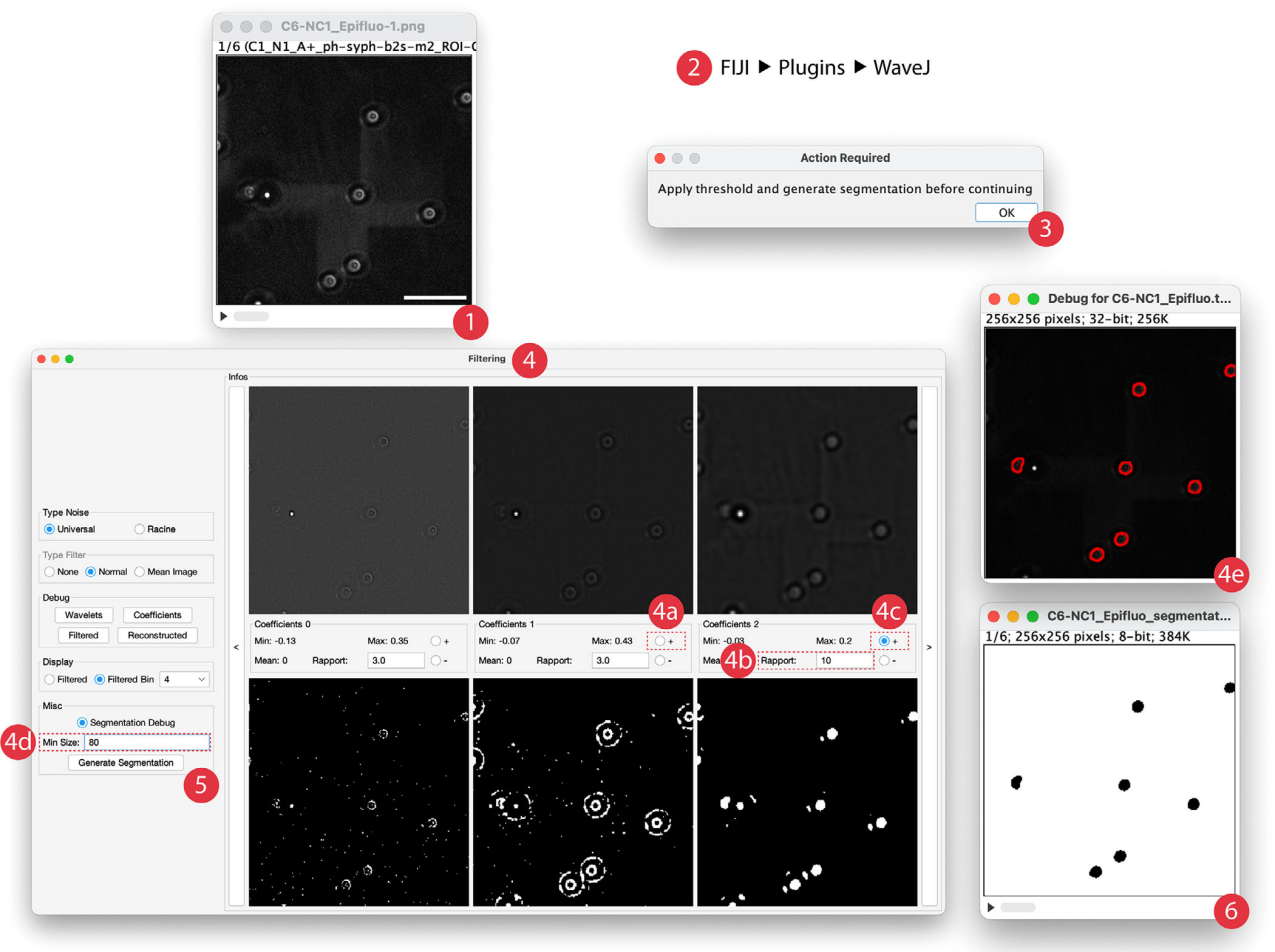
Epifluorescence image segmentation using the WaveJ Fiji plugin. We start from the individual fluorescent channel we want to segment (channel 6, corresponding to beads in this case) (1) and open the WaveJ Fiji plugin (2). We get a dialog asking to apply a threshold and generate a segmentation, we can click OK (3) The WaveJ ‘Filtering’ panel (4) displays wavelet coefficients at different scales. The bottom row shows the resulting binarised masks for each scale. After deselecting default settings (4a) and choosing a threshold factor for a specific coefficient (4b) we choose to add all white pixel of that wavelet level from the final image (4c). We need to input the minimum area (in squared pixels) for the objects we want to segment, beads in this case (4d). The debug window shows and updated output representation as we are adjusting parameters (4e). Once we are done with the settings we click on ‘Generate Segmentation’ (5) to create the final binarised image (6), which is ready for export and downstream comparison. In the figure we present screenshots from Fiji. Scale bar  =  10 µm.

By using a custom‐made Python registration script (now available at https://github.com/flevet/globias_bia) we then (1) upsized by a factor of 4 the epifluorescence images and downsized by a factor of 10 the SMLM ROIs, bringing both images to a 40 nm pixel size and (2) created a RGB stack (included in https://doi.org/10.5281/zenodo.20386408, under *01_processed_images* and each replicate folder: *NC1_*, *NC2_* and *NC3_bead_synaptophysin_segmented.tiff* files) where we combined the information from the actin SMLM acquisition (channel 1 in RGB stack), with the ß2‐spectrin image from the epifluorescence stack (channel 2 in RGB stack), and the combined contours of beads (intensity 125) and synaptophysin (intensity 255) segmented from the epifluorescence images (channel 3 in the RGB stack) (Figure 1C, iv).^13^, ^25^ This stack was automatically obtained by extracting the crop coordinates encoded in the SMLM filename, allowing precise alignment within the larger field of view of the 3 other channels.

### 2.5 Annotation of structures of interest

Once we had the pseudo‐images that combined information from SMLM and fluorescence microscopy, we were ready to identify the distinct presynaptic actin nanostructures the authors had described in their original publication. For this, and after having discussed with the corresponding author of the original work, we used slightly more concise descriptions of the structures of interest as the basis for our annotation step: (a) *actin mesh*: a small cluster of actin opposed to the bead contact and within the synaptophysin (previously described as presynaptic marker) cluster, (b) *actin rails*: linear structures within the synaptophysin cluster, and (c) *actin corrals*: large actin clusters at the periphery or just outside of the synaptophysin cluster.

We started with each analyst from our working group separately annotating each ROI with what we understood to be actin mesh, actin rails and actin corrals (annotations can be found at https://doi.org/10.5281/zenodo.20386408, under *02_annotated_images/02_analysts_independent_annotations/* where we have a folder per analyst including their individual manual annotation files per replicate and ROI)(Figure 1C, v). We then compared our results and reached a consensus annotation per ROI (at https://doi.org/10.5281/zenodo.20386408, under *02_annotated_images/01_analysts_consensus_annotations/* where per replicate annotations are stacked in *.tif* files; folders including individual manual annotation files per replicate and ROI are also provided) (Figure 1C, vi). These consensus annotations were then compared with the original author‐provided annotations for each ROI. To avoid bias, we did not inspect those original annotations before generating our own. All original annotations are available at https://doi.org/10.5281/zenodo.20386408, under *02_annotated_images/00_Bingham_original_annotations* as per replicate‐stacked *.tif* files, provided in two versions: (i) the files exactly as shared by the original authors and (ii) a harmonised version in which the colour code has been adjusted to match the external analysts’ annotations – see Figure 4A – to facilitate comparisons)^13^ (Figure 1D).

**FIGURE 4:**
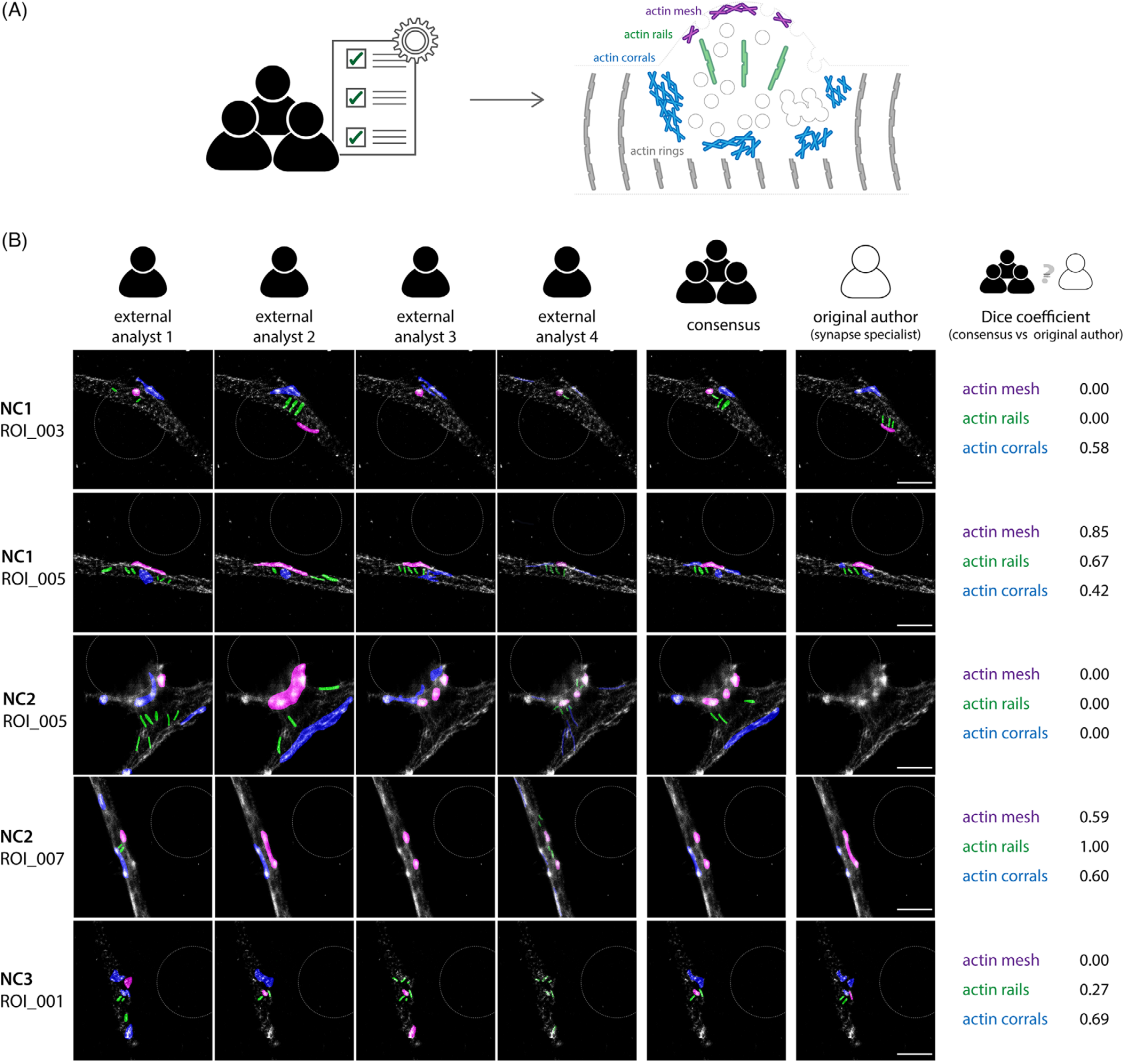
Actin nanostructure manual annotation. (A) We aimed at identifying and annotating the different types of actin nanostructures (actin mesh in magenta, actin rails in green and actin corrals in blue) described in Bingham *et al.* by following the bioimage analysis workflow described in the Method section of the original publication. The right part of the panel is adapted from Figure 10G from the original publication with kind permission from the authors.^5^ (**B)** Each analyst performed independent annotations (colour codes as in panel A) for 34 ROIs (bead position is indicated by a dotted circle). Individual annotations were generated independently and subsequently reviewed in a structured adjudication session. *Consensus* annotations were defined at the object level through comparison of candidate structures across analysts and agreement on their class assignment and ROI placement, and were then contrasted with the *original* annotations provided by Bingham *et al.* (performed by a synapse biology specialist). Annotation agreement between *consensus* and *original* annotations for each ROI was quantified by the Dice coefficient for each actin nanostructure class. Scale bar  =  1 µm.

### 2.6 Consensus annotation procedure

We (four analysts) independently annotated all ROIs prior to any group interaction and without access to the annotations of the other analysts or those of the original authors. These independent annotations were then compiled into a shared comparison table and reviewed ROI by ROI in a synchronous adjudication session. During this session, we compared our interpretations of the actin signal patterns within the multimodal pseudo‐images, taking into account signal density, morphology, relative position to the bead, and the presynaptic marker context as described in the original publication (Figure 1C, v).

Consensus was established at the object level rather than at the pixel level; that is, the group evaluated whether visually identifiable actin patterns corresponded to mesh, rails, or corrals, and agreed on the placement of ROIs accordingly. A final consensus annotation was generated de novo during this session by one analyst operating Fiji, incorporating decisions reached through group discussion. Decisions were guided by majority agreement, typically requiring agreement from at least three of the four analysts, although discussion generally continued until a shared interpretation was reached whenever possible (Figure 1C, vi).

Free‐text comments were recorded per image during the adjudication process to capture uncertainty, image quality concerns, and reasoning behind inclusion or exclusion of candidate structures. Importantly, consensus annotations were established solely based on the descriptions and example images provided in the original publication. Only after this process were the annotations provided by the original authors consulted for comparison (Figure 1D).

### 2.7 Quantitative comparison of actin nanostructure annotations

To quantitatively assess inter‐annotator agreement, we used custom Python scripts (available at https://github.com/paul‐hernandez‐herrera/2026_Presynaptic_Actin_Nanostructures_A_Reproducibility_Case_Study) to convert actin mesh, corral and rail ROIs into binary masks, which were saved as *.tif* files and visually inspected by overlying them on the original images (all available at https://doi.org/10.5281/zenodo.20386408, under *02_annotated_images/*).^26^ Annotation agreement was then quantified by computing pairwise Dice similarity coefficients between all available binary masks for each ROI and actin class, using the standard definition $\mathrm{Dice}\left(A,B\right)=\frac{2|A\cap B|}{|A|+|B|}$, with empty–empty comparisons assigned a value of 1 (Figures 4B and S1).

## 3 RESULTS

Here, we present the results of an exercise conducted for the first in‐person *GloBIAS Bioimage Analysts workshop* (November 2024, Gothenburg, Sweden) as part of the ‘Bioimage analysis workflows’ session, in which external analysts attempted to reproduce bioimage analysis workflows published in recent research articles. Our group was composed of image analysts and computer scientists (with only one member having formal biology training).

The workflow we followed in this case study (summarised in Figure 1) began by identifying a suitable bioimage analysis workflow to assess for reproducibility (Figure 1A). Our first assigned article, Wigbers *et al.* (*Nature Physics*, 2021) did not provide direct access to data or code. The manuscript included *Data Availability* and *Code Availability* statements indicating that both were available from the corresponding author upon reasonable request.^27^ We contacted the corresponding author but did not receive a response.

The second article, Sitarska *et al.* (*Nature Communications*, 2023), made source code for image analysis available through a GitHub repository (https://github.com/JakubCzuchnowski/Supporting‐data‐for‐Sensing‐their‐plasma‐membrane‐curvature‐allows‐migrating‐cells‐to‐circumvent‐o).^28^ Sample image data, however, were not directly accessible. In line with the *Data Availability* statement, we contacted the corresponding author and received a swift and positive reply indicating a willingness to share the original data. Nonetheless, several practical constraints (including the corresponding author's travel commitments, the first author's relocation and lack of direct access to the original data, and the storage of large raw files on tape backups requiring time‐consuming retrieval) impeded timely access to the data. To ensure that we could complete the assignment within schedule for the *GloBIAS Bioimage Analysts Workshop* we were assigned a third article.

The third article, Bingham *et al.* (*Journal of Cell Biology*, 2023) provided access to analysis code via a GitHub repository (https://github.com/cleterrier/ChriSTORM), which enabled reproduction of the STORM image reconstruction.^5^ Sample image data were not directly available, and the repository did not include code or documentation for downstream annotation or ROI‐based classification. The *Data Availability* section indicated that data were available from the corresponding author upon reasonable request. After contacting them, we received a prompt reply and assurance that we would be given access to original image data for our analysis.

As a first step to assess the bioimage analysis workflow presented in the original work, we compared it with the community‐developed checklists published by Schmied *et al.* (Nature Methods, 2024), together with their online version (https://quarep‐limi.github.io/WG12_checklists_for_image_publishing/analysis_workflows/intro_analysis_workflows.html), an initiative from Quality Assessment and Reproducibility for Instruments & Images in Light Microscopy (QUAREP‐LiMi) to support the reproducibility of bioimage analysis workflows used in life science publications^2^ (Figure 1B). The results of this assessment, in which the original publication scored 0.5 out of 9 points, are summarised in Table 1 and indicate that the reporting of the analysis workflow did not meet the level of detail recommended by the community‐developed checklists. Consequently, reproducing the original analysis using only the *Methods* described in the original work and the publicly available information was not possible.

**TABLE 1:** QUAREP‐LiMi WG12 Checklist applied to Figure 7 of Bingham *et al.* (2023).

Checklist Item	What the guideline requires	How Bingham *et al.* (2023) performed	Compliance level (✓/⚠/✗)	Notes and implications for reproducibility
1. Cite components and platform	Explicit citation of the full analysis workflow/software/environment.	Image reconstruction is attributed to ThunderSTORM (Ovesný 2014) and ‘custom ImageJ macros (ChriSTORM)’ by the corresponding author, but these are not cited as a citable software objects and no version is specified. Fiji is cited.	⚠ Partial	A clear, stepwise description of the analysis workflow and the software required at each step is missing, making the reproduction of the workflow very challenging.
2. Key settings	Report variable parameters that affect outcomes (e.g., pixel size, thresholds).	No parameter values are provided in the publication.	✗	Lack of parameter reporting prevents precise replication of the analysis.
3. Example data	Provide test dataset to validate workflow.	No sample data included; the manuscript states that data are ‘available upon request’.	✗	Following our request, we gained access to the raw data (34 ROIs), which made the reproducibility assessment possible.
4. Manual ROIs	Include manually selected regions as separate files.	Manual annotations were performed by authors but were neither deposited nor referenced.	✗	After our request, we received one example set of ROIs. However, documentation explaining why those ROIs were considered valid was missing.
5. Exact version	Report version of code or commercial software.	No version identifiers are reported for ImageJ, ThunderSTORM, or ChriSTORM.	✗	Absence of version information makes difficult to determine whether software updates could alter results.
6. All settings (recommended)	Provide or export full configuration file or macro list.	Not supplied.	✗	Without complete settings, reproducing the exact reconstruction and analysis is not feasible.
7. Public example (recommended)	Make an example dataset publicly accessible.	No example database is deposited.	✗	All replication attempts depended on personal communication with the authors.
8. Document usage (ideal)	Show screen captures/videos/tutorials.	No such documentation is provided.	✗	A limited number of example structures are shown in the paper, but their definitions are not clearly explained for non‐biologists.
9. Cloud container (ideal)	Provide containerised/cloud‐hosted reproducible environment.	None provided.	✗	The absence of containerised environment increases the effort required from others to reconstruct a comparable setup.

*Note*: The assessment gives an overall score of 0.5/9, with 0.5/5 for minimal requirements (1–5), and 0/2 for both recommended (6–7) and ideal (8–9) features. See checklist for details.

The segmentation of presynaptic actin nanostructures presented in Figure 7 of the original publication had been  manually performed following visual inspection of the images.^5^ In the *Methods* section, the authors provided a description of the criteria followed to identify the nanostructures, with some of the described features (e.g. presynaptic marker cluster) being difficult to identify for non‐specialists in neuron biology. Given the limited number of sample images presented in Figure 7 and the lack of access to sample image data, we contacted the authors to obtain the raw image data and to clarify in more detail the criteria and steps used to identify the actin nanostructures (Figure 1C, i).

Starting from localisation coordinate files in .csv format, we used the ThunderSTORM plugin in Fiji for SMLM image reconstruction (Figures 1C, ii and 2).^17^ We were able to reproducibly reconstruct and visualise super‐resolution images that were comparable to those provided by the original authors (available at https://doi.org/10.5281/zenodo.20386408, under *00_raw_images* in the *Recs_TS_ROIs* folders for each replicate).^13^

For the segmentation of synaptophysin and bead markers in our epifluorescence images (Figures 1C, iii and 3), we used WaveJ, a newly developed update of the wavelet component of SpineJ that extends the original plugin to handle image stacks and is now publicly available at https://github.com/flevet/WaveJ.^22^ Once these structures were segmented, we used a custom‐made Python script (available at https://github.com/flevet/globias_bia) to perform automatic registration of the SMLM and epifluorescence images and generate a pseudo‐image for each ROI, combining information from the SMLM reconstructions, the axon marker ß2‐spectrin, and the segmentations of presynaptic marker cluster synaptophysin and the presynaptic‐inducing beads (Figure 1C, iv).^25^ Given that the description used to identify the different actin nanostructures (see *Methods* for definitions) relies on the positions of both synaptophysin and beads as references, it would have been important to quantify how accurate our marker segmentation was. Unfortunately, we did not have access to corresponding images from the original authors, making it impossible to assess the reproducibility of these steps or to evaluate the impact that potential segmentation inaccuracies could have on the final annotations.

Next, each analyst independently annotated the ROIs without access to the annotations of other analysts or those of the original authors (Figure 1C, v). The per‐replicate distributions of annotated actin nanostructures identified by each analyst are summarised in Table 2. Strikingly, although the raw number of annotated structures varies between external analysts (from 108 to 261 total structures), the fractions of annotated mesh (18%–23%), rails (46%–55%), and corrals (28%–34%) are notably similar across analysts, indicating consistent classification of actin nanostructure types despite differences in overall annotation yield.

**TABLE 2:** Per‐analyst distribution of annotated actin nanostructures.

External analyst	Replicate	Mesh (*n*)	Mesh (fraction)	Rails (*n*)	Rails (fraction)	Corrals (*n*)	Corrals (fraction)	Total (*n*)
Analyst 1	NC1	9	0.19	25	0.53	13	0.28	47
	NC2	15	0.18	40	0.47	30	0.35	85
	NC3	22	0.17	78	0.60	29	0.22	129
	*Grand total*	**46**	**0.18**	**143**	**0.55**	**72**	**0.28**	**261**
Analyst 2	NC1	10	0.18	28	0.49	19	0.33	57
	NC2	16	0.34	18	0.38	13	0.28	47
	NC3	25	0.18	72	0.53	40	0.29	137
	*Grand total*	**51**	**0.21**	**118**	**0.49**	**72**	**0.30**	**241**
Analyst 3	NC1	8	0.18	24	0.55	12	0.27	44
	NC2	6	0.14	21	0.49	16	0.37	43
	NC3	16	0.21	32	0.43	27	0.36	75
	*Grand total*	**30**	**0.19**	**77**	**0.48**	**55**	**0.34**	**162**
Analyst 4	NC1	9	0.21	25	0.58	9	0.21	43
	NC2	5	0.19	13	0.50	8	0.31	26
	NC3	11	0.28	12	0.31	16	0.41	39
	*Grand total*	**25**	**0.23**	**50**	**0.46**	**33**	**0.31**	**108**

*Note*: Counts and fractions of actin structures annotated by four external analysts (Analysts 1–4) across three biological replicates (NC1–NC3). For each analyst–replicate combination, the table reports the number of actin mesh, rails, and corrals, and the total number of segmented nanostructures. Fractions for each structure type are defined as the count of that structure divided by the total number of segmented structures in the corresponding analyst–replicate row.

As described in the *Methods* section, these annotations were subsequently reviewed in a structured consensus adjudication session to generate a single consensus annotation per ROI (Figure 1C, vi). This process revealed substantial variability in how the textual criteria from the original publication were interpreted. While analysts often identified similar regions of increased actin signal, disagreements frequently arose regarding whether these regions satisfied the definitions of mesh, rails, or corrals, particularly in cases where signal density and spatial context were ambiguous.

When trying to reach a consensus annotation, we realised that there were many inconsistencies in the results obtained by the different analysts. We soon noticed that there were slight differences in the way each analyst interpreted the author's description, which affected the annotations (e.g. while one analyst interpreted the definition of *mesh* nanostructure as ‘single brightest actin spot near bead’, another one considered it as ‘bright cluster inside the synaptophysin segmentation’). These discrepancies highlight that purely qualitative definitions (e.g. ‘small’ versus ‘large’ actin clusters to distinguish mesh from corrals) are insufficient for reproducible annotation, and suggest that future work should establish more objective, quantitative criteria – such as size ranges or relative area thresholds with respect to the synaptophysin cluster – that are easily interpretable by non‐experts.

When comparing our results with those from the original publication (Figure 1D, and for representative examples, see Figure 4B and at https://doi.org/10.5281/zenodo.20386408, under *02_annotated_images/* where annotated stacked *.tif* files can be compared between *00_Bingham_original_annotations* and *01_analysts_consensus_annotations* folders for all ROIs), there were examples where there was a clear consensus among the analysts and the annotation nicely fitted with the one generated by the authors (Figure 4B, NC1, ROI_005). In other cases, reaching a consensus annotation within the analyst group was more challenging, but in the end, the consensus was also in agreement with the authors’ annotations (Figure 4B, NC3, ROI_001). Strikingly, there were cases in which the analysts easily agreed on a consensus that nevertheless entirely differed from the authors’ annotation (Figure 4B, NC2, ROI_005, which had been left unannotated). When we consulted the author's notes for these ROIs, we realised that they had discarded specific ROIs based on assessments such as ‘the neuron did not look healthy’ or ‘the synapse had an odd shape’. These more qualitative, domain‐specific criteria – used as the basis for leaving some images unannotated – were not obvious to our working group (composed primarily of image analysts and computer scientists), and illustrate that explicit, upfront criteria for neuronal fitness and synapse morphology should be incorporated into the analysis workflow as a step prior to annotating presynaptic nanostructures. Another systematic difference between our annotations and those provided by the original authors was the tendency of our group to annotate structures even when confidence was low. In contrast, the original annotations frequently left ROIs unlabelled in images where no clear structures were identified. This difference in annotation threshold underscores the importance of explicitly defining criteria not only for when a structure should be annotated, but also for when an ROI should remain unannotated.

To provide a quantitative comparison between the reproduced annotations and the original author‐provided annotations, we calculated the Dice coefficient for each ROI and annotation class. Dice ranges from 0 to 1, where 1 indicates complete spatial overlap and 0 indicates no overlap. Overall, the Dice analysis supports the qualitative interpretation: agreement with the original annotations was limited and varied between structure classes (Figures 4B and S1). The consensus annotations generally provided a more stable representation than individual annotations, but they still did not fully recover the original annotations. These results reinforce that the main reproducibility limitation was not only boundary placement, but also interpretation of the qualitative criteria used to decide whether a structure should be annotated as mesh, rails, or corrals.

In all, our attempt to reproduce the actin nanostructure classification presented in Bingham *et al.* was partially successful. We were able to identify actin nanostructures resembling the three categories described in the original work (Table 2), and some of our annotations overlapped with those of the original authors (Figure S1). It is important to note that the comparison with the original study was limited by the availability of annotations from only a subset of the original analysts, rather than the consensus annotations described in the original publication or the images presented in the original Figure 7. As a result, discrepancies between our consensus and the available annotations may reflect differences in individual interpretation rather than disagreement with the original study's internal consensus.

## 4 DISCUSSION

In this work, we evaluated the reproducibility of a published bioimage analysis workflow within the framework of the first in‐person GloBIAS bioimage analysis workshop (2024, Gothenburg, Sweden). Our case study focused on the manual annotation of presynaptic actin nanostructures described in Bingham *et al.* (2023) and was carried out by external analysts with experience in bioimage analysis but without domain expertise in synapse biology. Our aim was to recreate the published annotations and to identify which aspects of data and workflow sharing most strongly influence reproducibility in practice. In doing so, we encountered several challenges and limitations, which we summarise below together with concrete recommendations for how authors and the broader community can improve the reproducibility of image analysis workflows.

A first major challenge was gaining access to representative image data and analysis code. The original study stated that data and code would be made available upon request, a practise that has since been highlighted as inefficient by community recommendations.^2^ In our case, several rounds of correspondence were required to obtain the SMLM localisation files, the corresponding epifluorescence images, and clarifications about the nanostructure definitions and workflow details. The original authors were highly supportive and invested considerable effort in supplying what we needed. Because we wished to publish our annotations, we also had to arrange open access to images we had not generated ourselves. With the authors’ collaboration and commitment to open science, these images were deposited on Zenodo (https://doi.org/10.5281/zenodo.20386408).^13^ Our experience illustrates why ‘*available upon request*’ and reliance on personal communication are time‐consuming and often inefficient. In line with current guidelines, and despite the effort and time investment required from authors, we recommend providing direct, public access to sample image data and code at the time of publication, accompanied by minimal documentation on how to reproduce analysis workflows.

A second difficulty concerned the way presynaptic actin structures were defined and annotated in the original work. The nanostructures of interest (actin mesh, rail, and corral) were described qualitatively and annotated manually based on expert visual interpretation. Fully automated workflows with fixed parameters are inherently easier to reproduce, but in many biological applications (particularly for SMLM data at very high spatial resolution) complete automation is not realistic. From the original study, some code was available via GitHub and the plugins used (ChriSTORM and ThunderSTORM) supported localisation processing and visualisation. However, because critical steps such as preprocessing, image registration, and annotation were not fully described, the shared code did not, on its own, allow us to reproduce the identification and quantification of presynaptic structures. In situations where full automation is not feasible, it becomes especially important to document manual steps and decision rules as explicitly as possible.

As external analysts without synapse biology expertise, we found it challenging to translate the textual definitions of mesh, rail, and corral into consistent annotation criteria, leading to substantial inter‐analyst variability and the need for a consensus process. Analysts often agreed on the presence of prominent actin patterns but differed on how they should be classified. Moreover, some images were excluded in the original study based on subtle indicators of neuron health that we could not reliably assess. These observations highlight that qualitative descriptions alone are insufficient to ensure reproducible interpretation of complex biological structures. To improve reproducibility, qualitative labels could be complemented by approximate quantitative criteria, for example, typical size ranges in nanometres or pixels, aspect ratios, or distances to a reference marker, so that categories become more operational and less dependent on implicit expert judgement. It would also be helpful to share intermediate analysis outputs – such as overlay images, segmentation masks, or example ROIs for each class – so that others can follow how raw data evolve into final annotations and benchmark their own pipelines at multiple stages. In our case, the dataset size was constrained by the difficulty of acquiring data, limiting the diversity of examples and contributing to differences in annotation outcomes.

Moreover, the task was inherently interpretative and object‐based (rather than a pixel‐wise segmentation problem). Quantitative overlap metrics such as Dice or intersection‐over‐union could in principle be computed from the deposited annotation masks, but they would not fully reflect disagreements that primarily concern object classification and interpretation rather than precise boundary delineation. Annotation schemes should therefore also specify under which conditions ROIs are left unannotated or excluded (e.g. low signal‐to‐noise ratio, ambiguous morphology, or incomplete marker information), as these decisions substantially influence downstream analyses.

Because no single researcher typically has all the required expertise, we see clear benefits in collaborative workflows that bring together domain specialists (such as synapse biologists) and image analysts throughout a project, so that aspects from biological design to algorithm implementation are adequately addressed. In this case, the original paper was written primarily from a biological perspective, with limited emphasis on enabling independent reproduction of the image analysis. This highlights the need for stronger communication between the bioimage analysis and biology communities and/or for better dissemination and implementation of existing recommendations for reproducible practices. Although such guidelines are generally straightforward to implement, they can seem abstract to researchers who are not yet familiar with their terminology or purpose. We believe that these practises would be adopted more consistently if their relevance were illustrated with concrete, domain‐specific examples, such as the case study we present here. Our exercise confirms that, with clear methodological steps and specified structural definitions, some level of reproducibility is achievable even for manual analyses, and that collaborative discussion (particularly with domain specialists) can greatly improve interpretation.

Ultimately, our attempt to reproduce a bioimage analysis workflow reinforces the importance of open access to sample data, methods, code, and intermediate analysis steps, and it highlights the value of involving dedicated bioimage analysis expertise alongside biological expertise. Rather than serving as a criticism of a specific publication, this exercise functioned as a structured learning activity: analysts from different backgrounds worked together in a workshop setting to recreate annotation following a published bioimage analysis workflow, negotiate ambiguous definitions, and document gaps that hinder reproducibility. We also note that the original work by Bingham *et al.* (2023) was published before the current community‐developed checklists by Schmied *et al.* (2024), which underscores both the value of these guidelines and the need to monitor their adoption (e.g. through similar reproducibility exercises, surveys at bioimage analysis workshops, or systematic reviews of recently published image analysis workflows). Our work aligns with the broader initiative articulated by Schmied *et al.* to develop educational materials and tutorials based on community checklists. By openly describing both obstacles and potential solutions, our study offers a case‐based resource. We see substantial value in similar ‘reproducibility challenges’ and think that implementing them as a regular practise in teaching or training events could help normalise good practices and provide concrete examples of how community guidelines can inform the design, sharing, and evaluation of bioimage analysis workflows.

## AUTHOR CONTRIBUTIONS

Analysis: RP Theart, F Levet, P Hernández‐Herrera, LR de la Ballina. Experiments: C Leterrier. Methodology: RP Theart, F Levet, P Hernández‐Herrera, LR de la Ballina. Resources: C Leterrier. Software: RP Theart, F Levet, P Hernández‐Herrera, LR de la Ballina. Supervision: RP Theart, F Levet, P Hernández‐Herrera, LR de la Ballina. Validation: RP Theart, F Levet, P Hernández‐Herrera, LR de la Ballina. Visualisation: RP Theart, F Levet, P Hernández‐Herrera, LR de la Ballina. Figure preparation: LR de la Ballina. Writing – original draft: RP Theart, F Levet, LR de la Ballina. Writing – review & editing: RP Theart, F Levet, P Hernández‐Herrera, C Leterrier, LR de la Ballina.

## CONFLICT OF INTEREST STATEMENT

The authors declare no conflict of interest.

## Supporting information



Supporting Information

## Data Availability

Raw, processed and annotated images referred to in this work have been deposited in Zenodo and are publicly available at https://doi.org/10.5281/zenodo.20386408. Fiji plugins used as part of the analyses can be found at https://github.com/kjamartens/thunderstorm for ThunderSTORM and at https://github.com/flevet/WaveJ/releases/tag/1.0.0 for WaveJ. Python scripts can be found at https://github.com/flevet/globias_bia and https://github.com/paul‐hernandez‐herrera/2026_Presynaptic_Actin_Nanostructures_A_Reproducibility_Case_Study.^12^, ^13^, ^17^, ^22^, ^25^, ^26^
