# Supplementary material for: Presynaptic actin nanostructures: A reproducibility case study
Source: J Microsc. 2026 Jul 24;304(2):145–61. doi: 10.1111/jmi.70138 (PMC13686596; doi:10.1111/jmi.70138)
Supplement: Supplementary file 1 [file JMI-304-145-s001.docx]

**Supplementary Figure 1**. **Quantitative comparison of manual annotations using the Dice coefficient**. Dice coefficients were calculated between the original author-provided annotations and the annotations generated by the external analysts, including the final consensus annotations, for each ROI and actin nanostructure class: **A)** mesh, **B)** rails, and **C)** corrals. Dice values range from 0, indicating no spatial overlap, to 1, indicating complete overlap. Empty-empty comparisons were assigned a Dice value of 1. The plots show that overlap with the original annotations was variable across ROIs and structure classes, consistent with the qualitative observation that differences arose from both spatial annotation differences and interpretation of the structure definitions.
